# Linking Activation of Microglia and Peripheral Monocytic Cells to the Pathophysiology of Psychiatric Disorders

**DOI:** 10.3389/fncel.2016.00144

**Published:** 2016-06-03

**Authors:** Yuta Takahashi, Zhiqian Yu, Mai Sakai, Hiroaki Tomita

**Affiliations:** ^1^Department of Disaster Psychiatry, International Research Institute of Disaster Science, Tohoku UniversitySendai, Japan; ^2^Department of Disaster Psychiatry, Graduate School of Medicine, Tohoku UniversitySendai, Japan; ^3^Department of Psychiatry, Graduate School of Medicine, Tohoku UniversitySendai, Japan; ^4^Group of Mental Health Promotion, Tohoku Medical Megabank Organization, Tohoku UniversitySendai, Japan

**Keywords:** microglia, monocyte, mental disorder, psychoimmunology, peripheral biomarker, blood-brain barrier, schizophrenia, bipolar disorder

## Abstract

A wide variety of studies have identified microglial activation in psychiatric disorders, such as schizophrenia, bipolar disorder, and major depressive disorder. Relatively fewer, but robust, studies have detected activation of peripheral monocytic cells in psychiatric disorders. Considering the origin of microglia, as well as neuropsychoimmune interactions in the context of the pathophysiology of psychiatric disorders, it is reasonable to speculate that microglia interact with peripheral monocytic cells in relevance with the pathogenesis of psychiatric disorders; however, these interactions have drawn little attention. In this review, we summarize findings relevant to activation of microglia and monocytic cells in psychiatric disorders, discuss the potential association between these cell types and disease pathogenesis, and propose perspectives for future research on these processes.

## Introduction

A variety of postmortem brain studies and recent positron emission tomography (PET)-based studies have identified microglial activation in psychiatric disorders, such as schizophrenia (Bayer et al., 1999; Radewicz et al., 2000; van Berckel et al., 2008; Tang et al., 2012; Fillman et al., 2013), bipolar disorder (Haarman et al., 2014; Hercher et al., 2014), and major depressive disorder (Torres-Platas et al., 2014). Several studies have also indicated an association between alterations in monocytic features and psychiatric disorders (Rothermundt et al., 1998; Theodoropoulou et al., 2001; Padmos et al., 2008; Drexhage et al., 2011).

By contrast, peripheral monocytes can differentiate into macrophages and dendritic cells in peripheral tissues, both of which share similarities with microglia in their cellular morphology and functions, such as phagocytic activities, the expression of cell surface markers and cytokine production, and similar gene expression profiles (Schmitz et al., 2009; Beumer et al., 2012; Shechter and Schwartz, 2013; Prinz and Priller, 2014). Under pathological conditions in brain disorders, monocytes are recruited from peripheral blood into the brain, where they cooperate with microglia in immune responses (Beumer et al., 2012; Shechter and Schwartz, 2013; Prinz and Priller, 2014). Considering the similarity and potency of the interactions between these two cell types, it is reasonable to speculate that correlations and interactions exist between the activation identified in microglia and peripheral monocytic cells in patients with psychiatric disorders, although these possibilities have drawn little attention. In this review, we summarize the accumulated findings regarding microglial and peripheral monocytic activation in psychiatric disorders and discuss the potential mechanisms linking microglial and monocytic activation with the pathogenesis of psychiatric disorders. Finally, we propose directions for future research on these potential associations.

## Accumulated Findings of Microglial Activation in Psychiatric Disorders

Postmortem brain studies have suggested an association between psychiatric disorders and microglial activation (Bayer et al., 1999; Radewicz et al., 2000; Tang et al., 2012; Fillman et al., 2013; Hercher et al., 2014; Torres-Platas et al., 2014). Regarding the qualitative assessment of microglial morphology, Hercher et al. (2014) observed that activated microglial cells were increased in prefrontal white matter from patients with schizophrenia, but not that from patients with bipolar disorder. Torres-Platas et al. (2014) showed that primed (activated) microglia were significantly increased compared with ramified (resting) microglia in the anterior cingulate cortices obtained from patients who died of depressed suicides compared with healthy controls, whereas the total densities of ionized calcium-binding adapter molecule 1 (IBA1) positive microglia did not differ between the depressed suicide cases and controls. Bayer et al. (1999) and Radewicz et al. (2000) reported that the expression of Human Leukocyte Antigen-antigen D Related (HLA-DR), which reacts with activated microglia, was increased in the frontal cortices of patients with schizophrenia in immunostaining studies. Fillman et al. (2013) demonstrated that the interleukin-6 (*IL-6*), *IL8*, and *IL1β* mRNA transcripts were over-expressed in the dorsolateral prefrontal cortex, and the density of major histocompatibility complex class II (MHC-II) receptor-positive microglia (i.e., antigen-presenting cells) was increased in the white matter of patients with schizophrenia. Tang et al. (2012) found positive correlations among several activated microglial markers in subjects with schizophrenia. They also showed that changes in the expression of genes that encode markers of activated microglia were associated with inflammatory markers in the arachidonic acid signaling pathway in patients with schizophrenia.

Recent PET-based evaluations of microglial activation may also be applicable to psychiatric patients (Veenman and Gavish, 2000, 2012; Papadopoulos et al., 2006; van Berckel et al., 2008; Doorduin et al., 2009; Takano et al., 2010; Haarman et al., 2014). PET radioligands, such as C-PK11195 and DAA1106, are selective for the 18 kDa translocator protein/peripheral benzodiazepine receptor, which is highly expressed in activated microglia and is involved in multiple cellular processes, such as apoptosis, the regulation of cellular proliferation, immunomodulation and steroidogenesis (Veenman and Gavish, 2000, 2012; Papadopoulos et al., 2006). van Berckel et al.’s (2008) PET study showed a significant increase in microglial activation in patients with schizophrenia who had a disease onset within 5 calendar years compared with controls, although all patients were under treatment with atypical antipsychotics, and confounding effects of drug treatments remain unexcluded. Interestingly, Doorduin et al. (2009) in a PET study of patients who had recovered from psychosis, observed no significant microglial activation. Although Takano et al. (2010) found no significant difference between [^11^C]DAA1106 binding in normal controls and patients with schizophrenia, the patients exhibited positive correlations between cortical [^11^C]DAA1106 binding, positive symptom scores and duration of illness. Haarman et al. (2014) observed a significant increase in ^11^C-R-PK11195 binding potential in the right hippocampus of patients with bipolar disorder type I compared with healthy controls.

## Microglial Function and Potential Mechanisms Underlying the Pathogenesis of Psychiatric Disorders

Microglia comprise ~12% of cells in the central nervous system (CNS; Vaughan and Peters, 1974); these cells are not uniformly distributed (Schmitz et al., 2009). More microglia are located close to neurons in the gray matter, with the highest concentrations in the hippocampus, olfactory telencephalon, basal ganglia, and substantia nigra (Lawson et al., 1990).

Accumulating evidence from fate-mapping studies suggests that the origin of most microglia is not the bone marrow after birth but hematopoietic stem cells in the yolk sac in the early developmental stage (Lassmann et al., 1993; Ginhoux et al., 2010; Schulz et al., 2012; Kierdorf et al., 2013; Prinz and Priller, 2014). Novel transgenic approaches have shown clear differences in the cellular characteristics of microglia and macrophages in the brain (Goldmann et al., 2013; Parkhurst et al., 2013; Yona et al., 2013). In contrast to macrophages, microglia are long-lived and are not replaced by circulating peripheral monocytes under physiological conditions.

Microglia can be polarized into two subtypes: M1 and M2, responding to certain stimuli. For examples, lipopolysaccharide (LPS) or interferon-γ induces polarization into M1, whereas IL-4 or IL-13 induces M2 phenotype (Orihuela et al., 2016). Microglia contain two subtypes: M1 and M2. The M1 subtype is characterized by the production of pro-inflammatory cytokines, such as IL-1β, IL-6, IL-8 and tumor necrosis factor alpha (TNF-α; Barger and Basile, 2001; Boche et al., 2013). The M1 phenotype is activated by damage-associated molecular patterns, such as ATP, S100 molecules, histones, and heat shock proteins (Lu et al., 2014; Wiersinga et al., 2014). By contrast, the M2 phenotype is characterized by the production of anti-inflammatory cytokines, such as IL-10, insulin-like growth factor 1 (IGF-1), transforming growth factor beta (TGF-β), and neurotrophic factors (Ekdahl, 2012; Boche et al., 2013; Hu et al., 2015). The M2 phenotype is activated by cytokines, such as IL-4, IL-13 and IL-25 (Boche et al., 2013; Maiorino et al., 2013).

Activated microglia retract their cellular processes and transform from a ramified state into an ameboid morphology, in which they respond to external stimuli induced by various pathological conditions, such as trauma, infection, or other damage to brain tissue (Marshall et al., 2013). Activated microglial functions include phagocytosis and the production and release of cytokines, reactive oxygen species and nitrogen species (Barger and Basile, 2001; Takaki et al., 2012; Réus et al., 2015). They express a profile of cell surface marker expression that is similar to that of other mononuclear phagocytes (specifically macrophages), such as cluster of differentiation 14 (CD14), MHC molecules and chemokine receptors (Rock et al., 2004). Activation of microglia under pathological conditions in the brain may exert neuroprotective effects by reducing protein aggregates; however, they may exert cytotoxic effects by secreting neurotoxic factors (Streit et al., 1999; Schmitz et al., 2009).

## Peripheral Monocytic Activation in Psychiatric Disorders

Several studies have suggested an association between monocyte activity and psychiatric disorders (Rothermundt et al., 1998; Theodoropoulou et al., 2001; Nikkilä et al., 2014). Some studies have shown that circulating peripheral blood monocytes are increased in patients with schizophrenia (Rothermundt et al., 1998; Theodoropoulou et al., 2001). In addition, in the cerebrospinal fluid of patients with schizophrenia, the numbers of monocytes and macrophages were increased during acute psychotic episodes (Nikkilä et al., 2014). In contrast to schizophrenia, the number and level of CD14-positive monocyte differentiation were not altered in patients with bipolar disorder compared with healthy controls (Padmos et al., 2008; Drexhage et al., 2011). Recently, Drexhage et al. (2010) conducted a series of gene expression profiling studies using monocytes from psychiatric patients (27 schizophrenia and 56 bipolar patients) and matched controls via a microarray analysis, followed by quantitative polymerase chain reaction (PCR) studies for validation (Padmos et al., 2008; Beumer et al., 2012). The authors identified two main subsets of strongly correlated genes: one subset was composed of pro-inflammatory cytokines; the other subset consisted mainly of adhesion/motility factors. The monocyte gene expression profiles of the majority of the patients with bipolar disorder showed dysregulation in both subsets, whereas the monocyte gene expression profiles of the majority of the schizophrenia patients showed dysregulation only in the subset of pro-inflammatory cytokines (Drexhage et al., 2010).

## Subpopulations of Peripheral Monocytic Cells and Potential Pathogenic Mechanisms Underlying the Involvement of Peripheral Monocytes in Psychiatric Disorders

Monocytes are precursors of tissue macrophages, osteoclasts, and antigen-presenting cells (Lawson et al., 1990; Schmitz and Grandl, 2007). Monocytes, which comprise 5–10% of peripheral blood leukocytes, are derived from myelomonocytic stem cells in bone marrow and then released into the circulation, where they have a half-life of up to 3 days in humans (Whitelaw, 1972; Fogg et al., 2006). The brain harbors several types of monocyte-derived cells (Prinz and Priller, 2014). Macrophages and blood-derived dendritic cells are both present in the outer boundaries of the brain, such as the choroid plexus, perivascular space, and meninges; however, the number of blood-derived dendritic cells is small (Prinz and Priller, 2014).

There are five subsets of monocytes; they can be distinguished by different surface markers (Schmitz et al., 1997; Stöhr et al., 1998; Rothe et al., 1999; Gordon and Taylor, 2005; Beumer et al., 2012). More than half of monocytes belong to subset 1 and are characterized by surface marker profiles with abundant CD14 and a lack of CD16 expression (Schmitz et al., 1997; Stöhr et al., 1998). Both subsets 2 and 3 have CD16 expression and comprise active phagocytic cells. The expression of CD14 is increased in subset 2 compared with subset 3 (Schmitz et al., 1997; Stöhr et al., 1998). Subset 4 is a precursor of dendritic cells with high expression of CD40 (Schmitz et al., 1997; Stöhr et al., 1998). Subset 5, the smallest subset, shares many surface markers with subset 1; however, it differs in the additional expression of CD56, a marker of immature monocytes (Schmitz et al., 1997; Stöhr et al., 1998). Transformation between subsets among peripheral monocytes can occur concomitant with the differentiation of microglia in the brain under certain pathogenic conditions related to psychiatric diseases, although these possibilities remain to be elucidated.

## Potential Mechanisms Linking Activation of Microglial and Peripheral Monocytic Cells to Psychiatric Disorders

**Figure 1:**
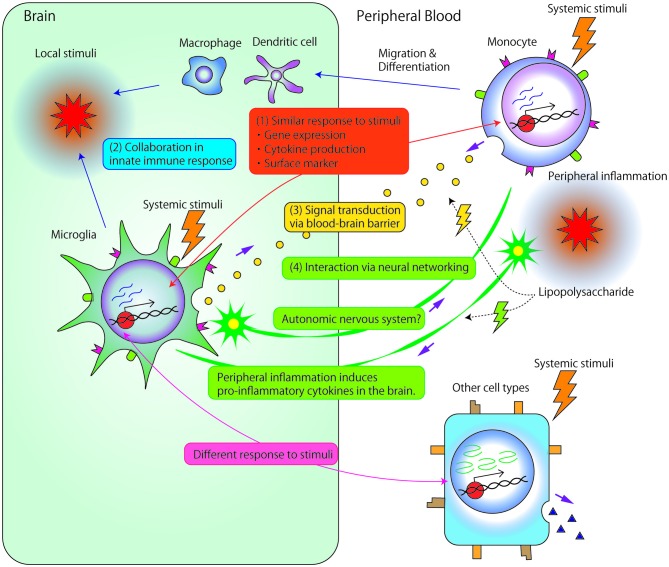
**Potential mechanisms underlying the associations and/or interactions between microglia and monocytes. (1)** Microglia and monocytes exhibit similar responses to systemic stimuli. First, accumulated data have suggested strong associations between microglia and monocyte gene expression. Second, the two cell types show similar profiles for cytokine production, such as interleukin (IL) 1β, IL-6, IL-8 or tumor necrosis factor (TNF)-α. Third, both cell types express similar surface markers, such as cluster of differentiation 14 (CD14), major histocompatability complex (MHC) molecules, and chemokine receptors. **(2)** Local stimuli in the brain facilitate the recruitment of circulating monocyte-derived macrophage/dendritic cells into the central nervous system (CNS). Immigrant monocytic cells, which are short-lived, and localized in the outer boundaries of the brain compared with microglia (Prinz and Priller, 2014), may collaborate with microglia in the innate immune response. **(3)** Signals are transduced between microglia and circulating monocytes through the blood-brain barrier (BBB). Even relatively large molecules, such as cytokines, may comprise vehicles of signal transductions through circumventricular organs brain lymphatic pathways, BBB-relevant transporters/receptors, or abnormal permeability of BBB. **(4)** Microglia and peripheral monocytic cells may exert related biological reactions through neuronal signal transduction. Peripheral inflammation, induced by, for example, the peripheral administration of lipopolysaccharide (LPS), induces reactions in peripheral monocytic cells and also triggers the production of pro-inflammatory cytokines in the brain. Such reactions in the brain are thought to be induced by the transduction of neural excitability from peripheral nerves to the CNS because LPS rarely penetrates the BBB. Additionally, interactions between microglia and neuronal networks modulate myeloid cell proliferation through the autonomic nervous system.

As previously described, many studies have investigated the association between microglia and psychiatric disorders or monocytes and psychiatric disorders. However, only a few studies have investigated the direct association or interaction between microglia and monocytes. Theoretically, there are several potential mechanisms underlying these interactions, as described below (Figure 1).

### (1) Common Responses of Microglia and Peripheral Monocytic Cells to Endogenous or Exogenous Stimuli

As mentioned in the introduction, microglia and monocytes have similar functions, such as phagocytosis and the release of pro-inflammatory cytokines, as well as similar expression of surface markers, such as CD14, MHC molecules, and chemokine receptors (Schmitz et al., 2009; Beumer et al., 2012; Shechter and Schwartz, 2013; Prinz and Priller, 2014). They may also share similar epigenetic marks, in addition to having the same genomic sequence. Although limited data directly indicate epigenetic similarities between microglia and peripheral monocytic cells, several previous studies have suggested similarities in the gene expression profiles of both microglia and peripheral monocytic cells. Schmitz et al. (2009) observed over-representation of genes associated with Alzheimer’s disease among the expression profiles specific to microglia and monocytes (Thomas et al., 2006; Lutter et al., 2008). The authors compared the microglial gene expression profile with the microarray data of human blood monocytes and *in vitro* macrophage colony-stimulating factor (M-CSF) differentiated macrophages to assess the expression of genes associated with Alzheimer’s disease. Of 379 Alzheimer’s disease-related genes, 159 were expressed in microglia, 198 in monocytes and 206 in macrophages. Of these genes, the expression of 128 Alzheimer’s disease-related genes were shared by microglia, monocytes and macrophages.

In addition, we compared the gene expression profiles of mouse microglia and monocytes in our preliminary microarray study prior to evaluating the effect of lithium treatment on microglia and peripheral monocytic cells (Yu et al., 2015). These findings also suggested a strong association between microglia and monocyte gene expression patterns. Among 45,281 transcripts on the microarray, 11,597 with average signal intensities greater than 100 in microglia or monocytes were reliably detectable. Of these transcripts, 7552 genes were identified in both cell types, including 2832 that were expressed only in microglia, and 1213 that were expressed only in monocytes. The correlation coefficients among the microglia samples were 0.94–0.98, compared with 0.97–0.98 for monocytes and 0.71–0.76 between microglia and monocytes. By contrast, the alterations in the gene expression profiles following lithium treatment were quite different between the microglia and other types of monocytic cells. Yu et al. (2015) investigated the effects of lithium on two cell types: monocyte-derived dendritic cells treated with lithium and microglia separated from lithium-treated mice. They compared the gene expression profiles of these cells with those of controls. They demonstrated that the common gene significantly induced by lithium in both monocyte-derived dendritic cells and microglia was only the third component of complement. Taken together, these findings indicate that microglia and monocytes have a similar gene expression pattern under normal conditions; however, changes in the pattern of expression profiles in response to stimuli are quite different.

### (2) Collaboration of Peripheral Monocytes with Microglia in the Innate Immune Response

Damage to the CNS commonly results in the recruitment of circulating immune cells, including monocytes, which results in an innate immune response that consists of microglia and monocyte-derived macrophages/dendritic cells (Prinz and Priller, 2014). The differential roles of these myeloid cell populations in CNS disorders have only recently been acknowledged and were nicely illustrated in a recent review by Prinz and Priller (2014). In Alzheimer’s disease, activated microglia have been associated with amyloid-β-induced neurotoxicity, and microglia were also damaged by amyloid species (Simard et al., 2006; Mildner et al., 2011; Prinz and Priller, 2014). Transplantation of wild-type bone marrow cells in transgenic mouse models of Alzheimer’s disease causes the migration of bone marrow-derived phagocytes to the brain after CNS preconditioning. Consequently, the amyloid load in the brain is eliminated by bone marrow-derived phagocytes (Simard et al., 2006; Mildner et al., 2011; Prinz and Priller, 2014). Similarly, recovery from spinal cord injury in mice has been reported to depend more on infiltrating monocyte-derived macrophages than on resident microglia (Shechter et al., 2009).

### (3) Interactions Between Microglia and Peripheral Monocytic Cells Through the Blood-Brain Barrier

Interactions between microglia and monocytes may also be regulated by cytokines. In a mouse model of amyotrophic lateral sclerosis, Butovsky et al. (2012) demonstrated that chemokine receptor-2 (CCR2) was over-expressed in splenic Ly6C^hi^ monocytes at disease onset, which was paralleled by upregulation of chemokine ligand-2 (CCL2) in CD39^+^ microglia. Additionally, CCR2 was not expressed in CD39^+^ microglia, and CCL2 was not expressed in Ly6C^hi^ monocytes during the pathological course of the disease. Therefore, the authors suggested that the expression of CCL2 and other chemokines on microglia caused the migration of Ly6C^hi^ monocytes to the CNS.

Furthermore, both microglia and monocytes are activated by and release pro-inflammatory cytokines, such as IL-1β, IL-6, IL-8 or TNF-α (Chan et al., 2007; Schmitz et al., 2009; Beumer et al., 2012). Studies have reported that these cytokines are increased in the blood of psychiatric patients (Naudin et al., 1997; Kim et al., 2000; Drexhage et al., 2008; Padmos et al., 2008; Song et al., 2009). Therefore, it is reasonable to suspect that these cytokine activation might underlie interactions between microglia and monocytes, although there has not been direct evidence to support the theory.

However, cytokines are relatively large molecules and rarely cross the blood-brain barrier (BBB). There are several known mechanisms and routes by which they can cross the barrier, such as alterations in the barrier’s permeability (Esposito et al., 2001; Stamatovic et al., 2006), crossing though circumventricular organs (Anisman, 2009; Calderó et al., 2009; Banks and Erickson, 2010), or the use of specific transporters or receptors (Kastin et al., 1999; Chesnokova and Melmed, 2002; Banks and Erickson, 2010). Also, recent studies discovered functional lymphatic vessels lining the dural sinuses which can convey fluid, large molecules, and even immune cells from the brain and are connected to the deep cervical lymph nodes. Further researches into the CNS lymphatic system may facilitate understanding of interactions between microglia and peripheral monocytic cells (Iliff et al., 2015; Louveau et al., 2015).

### (4) Interactions Between Microglia and Peripheral Monocytic Cells Through Neuronal Networking

Peripheral inflammation, which is caused, for example, by peripheral administration of LPS, induces peripheral reactions among immune cells, including monocytes. LPS also induces pro-inflammatory cytokines in the brain; however, LPS rarely crosses the BBB (Hayashi et al., 2015). This phenomenon may reflect that activation of inflammatory responses in the brain is induced by transduction of neural excitability from peripheral nerves to the CNS (Banks and Robinson, 2010), as well as LPS-induced dysfunction of vascular endothelial cells at the BBB (Banks et al., 2015). These findings suggest that mild peripheral inflammation, which contributes to the pathogenesis of psychiatric disease-related events, including fatigue, may induce microglial activation through neuronal networking.

Additionally, it is possible that interactions between microglia and neuronal networks modulate myeloid cell proliferation through the autonomic nervous system, although there is little evidence (Mignini et al., 2003; Spiegel et al., 2008). Peripheral monocyte activities may reflect microglial activities through these mechanisms.

## Perspectives on Studies Linking Activation of Microglial and Peripheral Monocytic Cells to Psychiatric Disorders

Several animal models are characterized by abnormalities in both immune system function and behavior (Amrani et al., 1994; Yirmiya, 1996; Bothe et al., 2005; Frenois et al., 2007; Fu et al., 2010). These models have enabled us to observe the activation of circulating monocytes and microglia in the brain and to investigate their influence on behavior. The non-obese diabetic (NOD) mouse spontaneously develops autoimmune diabetes, which is similar to the onset of type 1 diabetes in humans. Psychiatric diseases in humans have been associated with autoimmune diseases, such as type 1 diabetes and autoimmune thyroiditis (Kupka et al., 2002; Padmos et al., 2004; Eaton et al., 2010). Interestingly, abnormal behaviors have also been observed in NOD mice (Amrani et al., 1994; Bothe et al., 2005). This model is useful for investigating associations between microglial activation and monocytes in pathological processes, as well as gene-environment interactions in this association. In addition, ion channels have recently been recognized to play important roles in the immune response of neurological disorders (Eder, 2010). Local changes in cell osmolality enable monocytes to migrate and invade the CNS parenchyma, where they further differentiate into phagocytes under pathological conditions (Fuentes et al., 1995; Bennett et al., 2003; Mahad and Ransohoff, 2003; Schwab et al., 2007). Ion channels also have important roles in the process of microglia activation (Eder, 2010). Therefore, ion channel inhibitors are good candidates for therapeutic interventions to control immune responses in psychiatric diseases (Wulff et al., 2000; Reich et al., 2005; Rangaraju et al., 2009; Eder, 2010).

## Conclusion

Microglia and monocytes have similar functions, surface markers, and gene expression profiles. Both cell types release pro-inflammatory cytokines when activated in response to stimuli in the brain under various pathological conditions. Accumulating data also indicate interactions between monocytes and microglia. Research into these interactions may lead to new strategies to elucidate the pathogenesis of psychiatric diseases, as well as to develop peripheral biomarkers that reflect the pathological conditions in the brain, including microglial activation related to the progression or modulation of disease.

## Author Contributions

HT contributed to the design of the manuscript. YT and HT drafted the manuscript. All authors contributed to construction of the context, revision of the manuscript. All authors read and approved the final manuscript.

## Conflict of Interest Statement

The authors declare that the research was conducted in the absence of any commercial or financial relationships that could be construed as a potential conflict of interest. The reviewer MO and handling Editor declared their shared affiliation, and the handling Editor states that the process nevertheless met the standards of a fair and objective review.

## Abbreviations

CNS: central nervous system
LPS: lipopolysaccharide
NOD: non-obese diabetic.
